# Optimal postoperative delirium prediction after coronary artery bypass grafting surgery: a prospective cohort study

**DOI:** 10.3389/fcvm.2023.1251617

**Published:** 2023-12-08

**Authors:** Ying Ma, Dongxin Sui, Shaozhong Yang, Xiaomei Yang, Joseph Oldam, Jessica L. Semel, Zhihao Wang, Ningning Fang

**Affiliations:** ^1^ Department of Geriatric Medicine, Qilu Hospital (Qingdao), Cheeloo College of Medicine, Shandong University, Qingdao, China; ^2^ Department of Respiration, The Second Hospital of Shandong University, Jinan, Shandong, China; ^3^ Department of Anesthesiology, Qilu Hospital of Shandong University, Jinan, Shandong, China; ^4^ B.S. Neuroscience, Center for Research on Cardiac Intermediate Filaments, Johns Hopkins University School of Medicine, Baltimore, MD, United States; ^5^ Center for Research on Cardiac Intermediate Filaments, Johns Hopkins University School of Medicine, Baltimore, MD, United States; ^6^ Department of Geriatric Medicine, Qilu Hospital of Shandong University, Jinan, Shandong, China

**Keywords:** postoperative delirium, ACCI, OPCABG, ICU stay, prospective study

## Abstract

**Background:**

Postoperative delirium (POD) presents as a serious neuropsychiatric syndrome in patients undergoing off-pump coronary artery bypass grafting (OPCABG) surgery. This is correlated with higher mortality, cognitive decline, and increased costs. The Age-adjusted Charlson Comorbidity Index (ACCI) is recognized as an independent predictor for mortality and survival rate. The purpose of our study is to estimate the predictive value of the ACCI on the POD in patients undergoing OPCABG surgery.

**Methods:**

This prospective cohort study enrolled patients undergoing OPCABG surgery between December 2020 and May 2021 in Qilu Hospital. Patients were divided into the low-ACCI group (score, 0–3) and the high-ACCI group (score ≥4) according to their ACCI scores. The Confusion Assessment Method for the Intensive Care Unit (CAM-ICU) and CAM were used to diagnose POD within 7 days after surgery. The general, laboratory, and clinical data of the patients were recorded and collected. The characteristic ROC curve was applied to further assess the predictive value of the ACCI for POD in patients following OPCABG surgery.

**Results:**

A total of 89 patients were enrolled, including 45 patients in the low-ACCI group and 44 patients in the high-ACCI group. The incidence of POD was higher in the high-ACCI group than in the low-ACCI group (45.5% vs. 15.6%, *P *= 0.003). Multivariate logistic regression analyses showed that the ACCI (OR, 2.433; 95% CI, 1.468–4.032; *P *= 0.001) was an independent risk factor for POD. The ACCI accurately predicted POD in patients following OPCABG surgery with an AUC of 0.738, and the Hosmer–Lemeshow goodness of fit test yielded *X*^2 ^=^ ^5.391 (*P *= 0.145).

**Conclusion:**

The high-ACCI group showed a high incidence of POD. The ACCI was an independent factor associated with POD in patients following OPCABG surgery. In addition, the ACCI could accurately predict POD in patients following OPCABG surgery.

**Clinical Trial Registration:**

ClinicalTrials.gov, identifier CHiCTR2100052811.

## Introduction

As the aging of the population intensifies and the incidence of diabetes and obesity increases, the incidence of coronary heart disease (CAD) increases with each passing year. Despite the rapid development of percutaneous coronary stent implantation, coronary artery bypass grafting (CABG) remains the main surgical treatment for patients with multiple vessel lesions (1). The off-pump CABG procedure is more widespread and accepted with the development of instrumentation for the improvement of heart stabilization. The benefit of off-pump CABG (OPCABG) is the absence of an extracorporeal circuit (ECC) induced systemic inflammatory response and air embolism (2).

Postoperative delirium (POD) is an acute alteration in attention/cognition that mostly occurs 6%–56% of the time 2–5 days after cardiac surgery (3–5). It is well known that ECC has a significant impact on the occurrence of POD. However, the probability of POD in patients with OPCABG is still high and may be related to an increased risk of stroke induced by atherosclerosis or calcified embolism in patients with aortic calcification (6). The occurrence of POD is associated with increased mortality, increased hospital stay length, higher medical costs, and reduced cognitive function (4, 7, 8). Approximately 30%–40% of POD can be prevented (9); therefore, early identification and the control of reversible risk factors are key to preventing POD and reducing POD-related complications.

Several measurements have been developed to assess and grade the degree of comorbidities, including the Age-adjusted Charlson Comorbidity Index (ACCI). The ACCI covers multisystem diseases and weights the scores according to age and the severity of the disease, which is suitable for elderly patients with cardiovascular disease and multimorbidity (10). Heretofore, ACCI is mainly used to evaluate the long-term prognosis of patients undergoing non-cardiac surgery (11–13). Liu explored the relationship between the ACCI and POD and emphasized the predictive effect of the ACCI on POD in senior patients undergoing abdominal and thoracic surgery (14). The purpose of our study is to explore the predictive effect of the ACCI on POD in patients undergoing OPCABG surgery.

## Methods

### Study design and participants

**Figure 1:**
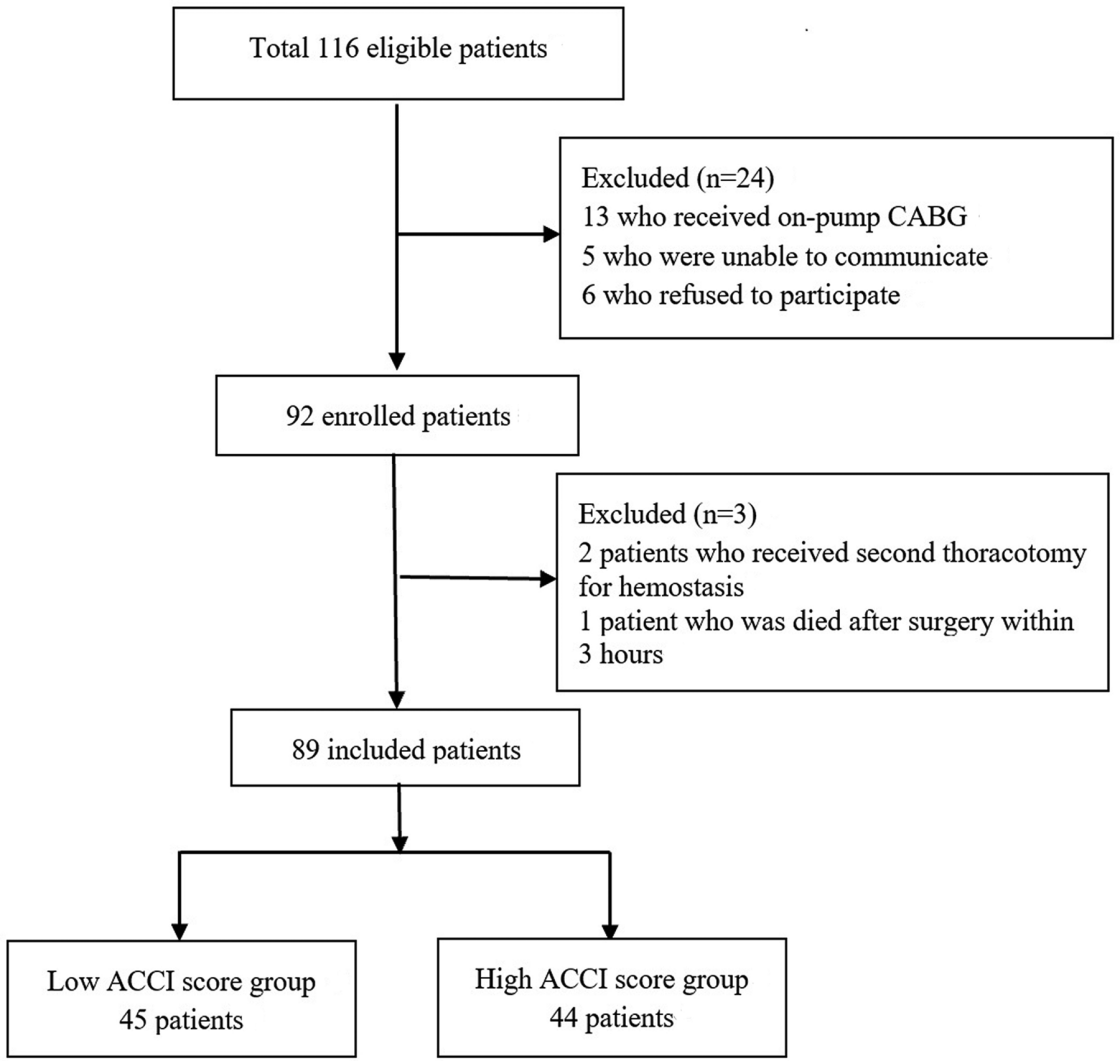
Flow chart of the study population.

This prospective study enrolled patients scheduled to undergo OPCABG surgery between December 2020 and May 2021 in Qilu Hospital of Shandong University. This study was conducted following the guidelines of the Consolidated Standards of Reporting Trials (CONSORT) and approved by Qilu Hospital of Shandong University Medical Ethics Committee (No. 2020-092). This study obtained written informed consent from all the participants and was registered at the China Clinical Trial Registry (CHiCTR2100052811). All procedures were carried out under the principles of the Helsinki Declaration. The recruitment and inclusion process are shown in Figure 1.

### Data collection

The demographic characteristics, laboratory, and clinical data, including gender, age, body mass index (BMI), American Society of Anesthesiologists (ASA) grade, left ventricular ejection fraction (LVEF), comorbidity, and laboratory tests were prospectively recorded and examined.

At admission, the ACCI score was collected as follows. (1) Each of the following diseases was counted as 1 point: myocardial infarction, congestive heart failure, cerebrovascular disease/transient ischemic attack, peripheral vascular disease, chronic obstructive pulmonary disease, peptic ulcer, mild liver disease, connective tissue disease, and diabetes without complications. (2) Each of the following diseases was counted as 2 points: dementia/Alzheimer's disease, hemiplegia, moderate-to-severe chronic kidney disease, non-metastatic solid tumors within 5 years, leukemia, lymphoma, and diabetes with complications. (3) The following disease was counted as 3 points: moderate-to-severe liver disease. (4) Each of the following diseases was counted as 6 points: human immunodeficiency virus (HIV) infection and metastatic solid tumors. (5) Age stratification score: <50 years old was 0 points, 50–59 years old was 1 point, 60–69 years old was 2 points, 70–79 years old was 3 points, and ≥80 years old was 4 points. Patients with CABG were often associated with a variety of comorbidities, and they were divided into two groups according to the ACCI score. Patients with mild (ACCI score: 0–1) and moderate (ACCI score: 2–3) comorbidities were grouped into the low-ACCI group (ACCI score: 0–3), whereas patients with severe comorbidities were grouped into the high-ACCI group (ACCI score ≥4) (15).

All patients received routine anesthesia during the cardiac surgery. The induction drugs used were midazolam 0.03 mg/kg, etomidate 0.2 mg/kg, sufentanil 1.5 ug/kg, and rocuronium 1 mg/kg. During the operation, rocuronium was pumped at a constant speed of 1 mg/kg/h, and sevoflurane was inhaled to maintain anesthesia and sufentanil 1 ug/kg was added during sternotomy. During the operation, the mean arterial pressure (MAP) was maintained at more than 70 mmHg, and the bispectral index (BIS) was maintained at 40–60. After surgery, we transferred all the patients to the ICU for further treatment. Clinical data included drug dosage, BIS, body temperature, hemodynamics data, duration of anesthesia, number of distal anastomoses, and the locations of those anastomosis. Hospital stay, ICU stay, postoperative hospital stay, and medical costs were recorded at discharge.

In our study, we used the Mini-Mental State Examination (MMSE) to assess the pre-operative cognitive condition (16). The Confusion Assessment Method-Intensive Care Unit (CAM-ICU) was used to evaluate POD during the mechanical ventilation period (17). CAM was used to assess the occurrence of POD after the patient could communicate normally. The occurrence of POD was determined twice daily (09–11 a.m. and 19–21 p.m.) within 7 days after surgery by a blinded professional researcher. At any timepoint, if the criteria for diagnosing POD were met, we defined it as POD.

### Outcomes

The primary outcome of our study was the incidence of POD in patients following OPCABG surgery. The secondary outcomes were extubation time, ICU stay, hospital stay, and medical cost.

### Sample size and statistical analysis

PASS 15 (NCSS, LLC Kaysville, UT, USA) was used to calculate the study sample size. Previous studies reported that the incidence of POD after cardiac surgery was 6%–56% (5). To detect this difference, we set the delirium incidence rate as 20% in the low-ACCI group and 50% in the high-ACCI group, at a two-sided significance level of 0.05, with 80% power. We estimated a sample size of at least 39 subjects in each group. To account for a 10% loss to follow-up, we had a recruitment goal of 87 patients.

SPSS 26.0 software (IBM, Armonk, NY, USA) was used to perform the statistical analysis. The grouping of patients was blinded to statistical analysts. The Shapiro–Wilk test was used to test the normal distribution of the continuous data, and normal distribution data were expressed as mean ± standard deviation. Continuous data with a skewed distribution were represented as median (IQR). Continuous variables were compared by one-way analysis of variance (ANOVA). Categorical data were presented as frequency (percentage) and the chi-square test was used to compare the differences. We performed a logistic regression to detect independent predictors of POD. Receiver operator characteristic curves (ROC) with area under the curve (AUC) were used to detect the accuracy. Hosmer–Limeshow and calibration curves were used to evaluate the calibration of the prediction model. A two-tailed *P* value <0.05 was considered statistically significant.

## Results

Initially, 116 patients undergoing OPCABG were included in our study of which 27 were excluded due to changing operation, poor communication, and second thoracotomy; therefore, 89 patients were included in the final analysis (Figure 1). Among them, 45 patients had ACCI scores of ≤3, and 44 patients had ACCI scores of ≥4. The high-ACCI group had a higher incidence of POD than the low-ACCI group (45.5% vs. 15.6%, *P *= 0.003). There were statistical differences in age, the incidence of postoperative delirium, and the incidence of comorbidities, including hypertension, diabetes, and unstable angina between the two groups (*P *< 0.05). Patients in the high-ACCI group had a longer extubation time (*P *= 0.003), ICU stay (*P *= 0.027), postoperative hospital stay (*P *= 0.014), and total hospital stay (*P *= 0.020) than the low-ACCI group. There were no statistical differences in the number and location of distal anastomoses between the two groups (Tables 1, 2**)**. In addition, the incidence of each comorbidity of the ACCI in both patient groups is shown in Table 3. Detailed laboratory findings at admission are listed in Supplementary Material Table S1. No significant differences were detected among the two groups (*P *> 0.05). The hemodynamics and application of inotropes during the perioperative period are shown in Supplementary Material Table S2.

**Table 1:** Comparison of the demographic and clinical data between two groups.

Characteristic	Total (*n* = 89)	Low-ACCI score group (*n* = 45)	High-ACCI score group (*n* = 44)	*P* values
Age, median (IQR), year	62.00 (54.00, 70.00)	55.00 (51.00, 62.00)	67.50 (62.25, 72.00)	**<0**.**001**
Male, no. (%)	67 (75.3)	33 (73.3)	34 (77.3)	0.807
BMI, mean ± SD, kg/m^2^	25.21 ± 3.28	25.33 ± 3.09	25.10 ± 3.50	0.743
SBP, median (IQR), mmHg	155.00 (139.00, 173.50)	147.00 (138.50, 165.00)	167.50 (144.75, 180.75)	0.010
DBP, median (IQR), mmHg	74.00 (66.00, 82.00)	75.00 (67.00, 84.50)	72.00 (66.00, 79.00)	0.443
HR, median (IQR)	75.00 (66.00, 85.00)	74.00 (66.50, 83.50)	75.50 (64.25, 89.75)	0.849
ASA grade, no. (%)				0.242
Ⅲ	87 (97.75)	45 (100.0)	42 (95.5)	–
Ⅳ	2 (2.25)	0 (0.0)	2 (4.5)	–
LVEF, median (IQR)	0.61 (0.55, 0.65)	0.60 (0.55, 0.65)	0.61 (0.53, 0.65)	0.811
Pre-operative MMSE scores, median (IQR)	26.00 (25.00, 27.00)	26.00 (25.00, 27.00)	26.00 (25.00, 27.00)	0.234
Comorbidities, no. (%)				
Hypertension	50 (56.2)	20 (44.4)	30 (68.2)	**0**.**033**
Diabetes	32 (36.0)	10 (22.2)	22 (50.0)	**0**.**008**
Unstable angina	29 (32.6)	7 (15.6)	22 (50.0)	**0**.**001**
Oral medication, no. (%)				
Calcium antagonists	39 (43.8)	16 (35.6)	23 (51.1)	0.137
β-blockers	37 (41.6)	16 (35.6)	21 (47.7)	0.286
Diuretics	11 (12.4)	3 (6.67)	8 (18.2)	0.118
Nitrates	38 (42.7)	18 (40.0)	20 (45.5)	0.671

Bold values indicate statistical significance. ACCI, age-adjusted Charlson comorbidity index; BMI, body mass index; SBP, systolic blood pressure; DBP, diastolic blood pressure; HR, heart rate; ASA, American society of anesthesiologists; LVEF, left ventricular ejection fraction; MMSE, mini-mental state examination.

**Table 2:** Comparison of detailed surgical records and postoperative information between two groups.

Variables	Total (*n* = 89)	Low-ACCI score group (*n* = 45)	High-ACCI score group (*n* = 44)	*P* values
Number of distal anastomoses, median (IQR) Location of distal anastomosis, no. (%)	3 (3.4)	3 (3.4)	3 (3.4)	0.446
Location of distal anastomosis, no. (%)				
Left anterior descending	87 (97.8)	44 (97.8)	43 (97.7)	0.987
Right coronary artery	86 (96.6)	45 (100)	41 (93.2)	0.075
Left circumflex artery	73 (82.0)	37 (82.2)	36 (81.8)	0.910
Diagonal branches or intermedius ramidus	39 (43.8)	20 (44.4)	19 (43.2)	0.992
Postoperative delirium, no. (%)	27 (30.3)	7 (15.6)	20 (45.5)	**0**.**003**
Duration of anesthesia, median (IQR), min	320.00 (276.50, 363.00)	320.00 (275.00, 350.00)	318.00 (285.00, 371.00)	0.843
Duration of anesthesia, recovery, median (IQR), min	200.00 (142.50, 300.00)	180.00 (140.00, 255.00)	204.50 (158.00, 330.00)	0.128
Extubation time, median (IQR), min	650.00 (475.00, 940.00)	540.00 (460.00, 705.00)	820.00 (542.00, 1,150.00)	**0**.**003**
ICU stay, median (IQR), day	2.00 (2.00, 3.00)	2.00 (2.00, 3.00)	2.00 (2.00, 4.75)	**0**.**027**
Postoperative hospital stay, median (IQR), day	13.00 (11.00, 15.00)	12.00 (11.00, 14.00)	14.00 (11.00, 18.75)	**0**.**014**
Hospital stay, median (IQR), day	22.00 (17.50, 28.00)	21.00 (17.00, 25.50)	23.50 (20.00, 29.75)	**0**.**020**
Medical costs, median (IQR), dollar	20,493.91 (18,467.32, 25,246.90)	20,228.95 (18,470.86, 23,751.11)	21,570.68 (18,057.85, 27,304.38)	0.148

Bold values indicate statistical significance.

ICU, intensive care unit.

**Table 3:** Comparison of the ACCI evaluation in patients with different ACCI scores.

	Participants, no. (%)		
Variables	Low-ACCI score group (*n* = 45)	High-ACCI score group (*n* = 44)	*P* values
Myocardial infarction	25 (55.6)	22 (50.0)	0.600
Congestive heart failure	5 (11.1)	10 (22.7)	0.143
Peripheral vascular disease	7 (15.6)	19 (43.2)	**0.004**
Cerebrovascular disease	0 (0.0)	2 (4.5)	0.148
Dementia	0 (0.0)	0 (0.0)	–
Chronic obstructive pulmonary disease	5 (11.1)	4 (9.1)	0.752
Connective tissue disease	0 (0.0)	3 (6.8)	0.075
Digestive ulcer disease	1 (2.2)	4 (9.1)	0.159
Diabetes (ordinary type)	6 (13.3)	17 (38.6)	**0.006**
Diabetes (with other organ damage)	1 (2.2)	7 (15.9)	**0.014**
Moderate or severe chronic kidney disease	0 (0.0)	2 (4.5)	0.148
Hemiplegic paralysis	0 (0.0)	0 (0.0)	–
Leukemia	0 (0.0)	0 (0.0)	–
Malignant lymphadenoma	0 (0.0)	0 (0.0)	–
Mild liver disease	2 (4.4)	0 (0.0)	0.157
Moderate liver disease	0 (0.0)	0 (0.0)	–
Severe liver disease	0 (0.0)	0 (0.0)	–
Solid tumor (no transfer)	0 (0.0)	0 (0.0)	–
Solid tumor (transfer)	0 (0.0)	0 (0.0)	–
Acquired immune deficiency syndrome (AIDS)	0 (0.0)	0 (0.0)	–
Age <50	8 (17.8)	1 (2.3)	**0.015**
Age (50–59)	23 (51.1)	3 (6.8)	**0.000**
Age (60–69)	12 (26.7)	19 (43.2)	0.102
Age (70–79)	2 (4.4)	21 (47.7)	**0.000**
Age (80–89)	0 (0.0)	0 (0.0)	–

Bold values indicate statistical significance.

The POD was significantly correlated with age, ACCI, pre-operative MMSE scores, extubation time, and ICU stay. Multivariate logistic regression showed that ACCI was independently correlated with POD (*β *= 0.889, *P *= 0.001) (Table 4).

**Table 4:** Univariate and multivariate logistic regression analyses of clinical associated risk factors for postoperative delirium (POD).

Variables	Univariate			Multivariate		
	*β*	RR (95% CI)	*P* value	*β*	RR (95% CI)	*P* value
Age	0.097	1.102 (1.035, 1.173)	**0**.**002**			
ACCI	0.889	2.433 (1.468, 4.032)	**0**.**001**	0.889	2.433 (1.468, 4.032)	**0.001**
Pre-operative MMSE scores	−0.459	0.632 (0.421, 0.949)	**0**.**027**			** **
Duration of anesthesia	0.272	1.313 (0.971, 1.776)	0.077			
Extubation time	0.027	1.027 (1.002, 1.053)	**0**.**033**			
ICU stay	0.403	1.497 (1.138, 1.969)	**0**.**004**			** **
Fibrinogen	−0.373	0.689 (0.385, 1.230)	0.207			
Serum albumin	−0.018	0.983 (0.864, 1.118)	0.790			
Total cholesterol	0.172	1.188 (0.770, 1.834)	0.437			
Low-density lipoprotein cholesterol	0.298	1.347 (0.747, 2.426)	0.322			
High-density lipoprotein cholesterol	0.612	0.543 (0.083, 3.526)	0.522			
Creatinine	0.006	1.006 (0.995, 1.017)	0.322			
BUN	0.105	1.111 (0.924, 1.335)	0.264			
Glucose	0.053	1.055 (0.766, 1.451)	0.744			
Calcium	0.345	0.708 (0.011, 45.734)	0.871			

Bold values indicate statistical significance.

**Figure 2:**
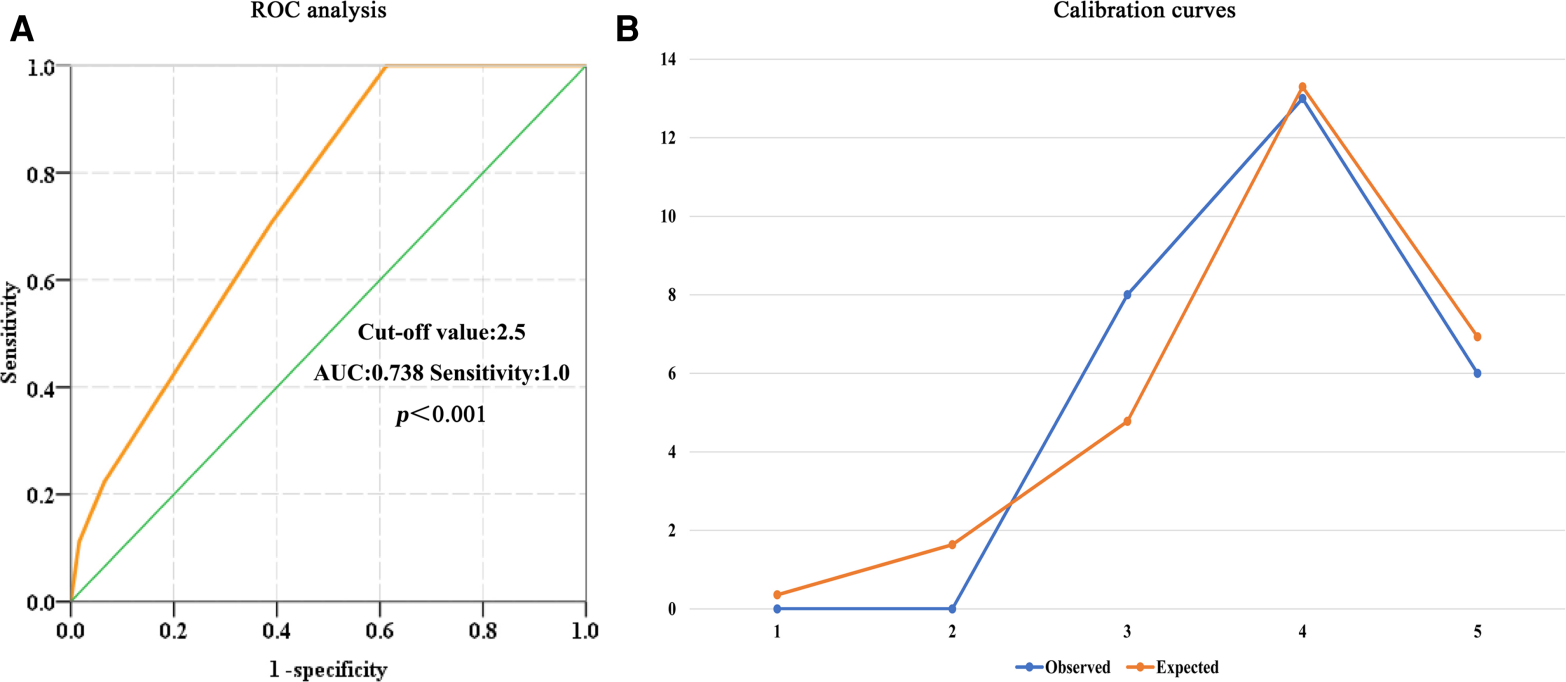
The predictive value of the ACCI for POD. (**A**) ROC analysis: the largest AUC is 0.738, the sensitivity of the ACCI is 1.0, and the optimal cutoff value of the ACCI is 2.5. (**B**) Calibration curves: the Hosmer–Limeshow goodness-of-fit test yielded *X*^2 ^= 5.391 (*P *= 0.145). ACCI, age-adjusted Charlson comorbidity index; POD, postoperative delirium; ROC, receiver operating characteristic; AUC, the area under the curve.

The characteristic ROC curve was applied to evaluate the predictive value of the ACCI for POD in patients following OPCABG surgery. Based on ROC analysis, the largest AUC was 0.738 and the sensitivity of the ACCI was 1.0. The optimal cutoff value of the ACCI was 2.5 for predicting POD in patients following OPCABG surgery, as shown in Figure 2A. In the Hosmer–Limeshow goodness-of-fit test, *X^2 ^*=*^ ^*5.391 (*P *= 0.145), indicating that there was no statistical difference between the predicted values of the model and the actual observed values. The validation results of the model were displayed in the calibration curve of the prediction model, as shown in Figure 2B.

## Discussion

Our prospective cohort study first revealed the predictive value of the ACCI for POD in patients following OPCABG surgery. Our study revealed that the high-ACCI group had a higher incidence of POD. Furthermore, our study indicated that the ACCI was independently associated with POD and can accurately predict POD in patients following OPCABG surgery.

Among all types of surgery, the incidence of POD occurred predominantly after cardiac surgery. In our study, the average occurrence of POD in patients following OPCABG surgery was 30.3%. The high-ACCI group had a significantly higher incidence of POD than the low-ACCI group (45.5% vs. 15.6%). The risk factors associated with POD included advanced age, preoperative co-morbidities, frailty, prolonged preoperative fasting, cardiopulmonary bypass time, depth of anesthesia, and medications during the perioperative period (18–20). We found that the POD was significantly correlated with age, the ACCI, pre-operative MMSE scores, extubation time, and ICU stay. For the controllable risk factors, optimization should be carried out during the perioperative period.

The ACCI was particularly well suited for classifying the comorbidities and estimating mortality from comorbid diseases (13, 21–23). Tseng et al. assessed the effect of the ACCI on the hospital survival of patients undergoing extracorporeal cardiopulmonary resuscitation (ECPR) and found that a high ACCI was correlated with a higher mortality rate (24). Zhang et al. investigated the correlation between the ACCI score and readmission/mortality in senior surgical patients (13). They demonstrated that the ACCI was independently associated with hospital readmission and all-cause mortality. The above studies mainly focused on the relationship between the ACCI and long-term prognosis in non-cardiac surgery. Liu et al. explored the correlation between the ACCI and POD and highlighted the predictive significance of the ACCI on POD in senior patients undergoing abdominal and thoracic surgery (14). They found that the ACCI was a sensitive predictive factor for POD, and the optimal intercept value of the ACCI was 5.5 according to ROC analysis. According to our research, the ACCI had high prediction sensitivity and specificity in patients following OPCABG surgery and the optimal cutoff value was 2.5. The reasons for this difference were the different study populations, the large trauma of cardiac surgery, the longer operation time, the higher dosage of opioid drugs during cardiac operation, the longer intubation, the impact of the ICU environment, and the inability of family members to accompany the patient after the operation.

The MMSE, which is a simple method for evaluating cognitive status, can identify POD early and deserved more attention as lower MMSE scores were typically associated with a higher risk of delirium (16, 25, 26). Segernäs found that pre-operative MMSE scores less than 27 were independently associated with a high risk of POD after cardiac surgery (16). Our finding was consistent with the results of this study. Our study detected that pre-operation MMSE was a negative factor associated with POD (*β *= −0.459, *P *= 0.027).

Previous studies have shown that POD is closely associated with a prolonged ICU stay (4, 27, 28). Patients with POD exhibited cognitive impairment and abnormal behavior, which were associated with an increased risk of bleeding, infection, and cerebrovascular accidents (4, 29). These complications increased ICU stay, the cost, and the length of rehabilitation. Our study confirmed a positive association between POD occurrence and ICU stay according to the results of logistic regression. Previous studies have shown that appropriate measures, such as earlier extubation, the promotion of patient mobility, pain control, POD prevention, nutrition, gastrointestinal function, and fluid management, can reduce the length of ICU stay (30).

There were several limitations in this study. First, this study was a single-center prospective study, and the number of cases involved was limited. Second, the ACCI was designed as a general predictor of mortality and outcomes. The ACCI might not capture nuances specific to OPCABG. In addition, the long-term follow-up results were not included. Further multicenter studies with large sample sizes are needed to verify the findings.

## Conclusion

In conclusion, the high-ACCI group had a higher incidence of POD, and the ACCI can accurately predict POD in patients following OPCABG surgery. The ACCI was independently associated with POD in patients undergoing OPCABG surgery. Appropriate risk stratification through the ACCI can manage POD through risk reduction measures and preventive interventions.

## Data Availability

The raw data supporting the conclusions of this article will be made available by the authors, without undue reservation.
