# Supplementary material for: Optimal postoperative delirium prediction after coronary artery bypass grafting surgery: a prospective cohort study
Source: Front Cardiovasc Med. 2023 Dec 8;10:1251617. doi: 10.3389/fcvm.2023.1251617 (PMC10739452; doi:10.3389/fcvm.2023.1251617)
Supplement: Supplementary file 1 [file Table1.pdf]

**Supplementary table 1** Detailed laboratory examinations at admission between two groups.

| Variables | Total(n=89) | Low-ACCI score group (n=45) | High-ACCI score group (n=44) | *P* values |
|---|---|---|---|---|
| Red blood cells, mean±SD, $10^{12}$/L | 4.43±0.60 | 4.47±0.49 | 4.38±0.70 | 0.470 |
| Hemoglobin, median (IQR), g/L | 138.00 (127.00, 146.00) | 138.00 (126.50, 146.00) | 137.50 (127.25, 145.75) | 0.917 |
| Platelet, median (IQR), $10^9$/L | 215.0 (177.00, 261.00) | 219.00 (176.00, 269.50) | 209.50 (177.00, 248.00) | 0.394 |
| Fibrinogen, median (IQR), g/L | 3.29 (2.73, 3.87) | 3.06 (2.73, 3.70) | 3.35 (2.70, 3.95) | 0.303 |
| Serum albumin, mean±SD, g/L | 41.26±3.50 | 41.17±3.24 | 41.35±3.79 | 0.813 |
| Total cholesterol, median (IQR), mmol/L | 3.81 (3.25, 4.57) | 4.00 (3.30, 4.72) | 3.57 (3.23, 4.22) | 0.128 |
| LDL- C, median (IQR), mmol/L | 2.16 (1.71, 2.80) | 2.37 (1.70, 3.10) | 2.04 (1.71, 2.49) | 0.135 |
| HDL- C, median (IQR), mmol/L | 1.07 (0.91, 1.24) | 1.09 (0.91, 1.33) | 1.03 (0.88, 1.21) | 0.304 |
| Triglycerides, median (IQR), mmol/l | 1.18 (0.90, 1.47) | 1.19 (0.90, 1.46) | 1.17 (0.95, 1.48) | 0.814 |
| Creatinine, median (IQR), µmol/L | 80.00 (69.00, 89.00) | 80.00 (72.00, 88.50) | 79.50 (66.00, 90.00) | 0.580 |
| BUN, median (IQR), mmol/L | 5.40 (4.70, 6.53) | 5.80 (4.95, 6.82) | 5.20 (4.53, 6.40) | 0.125 |
| Glucose, median (IQR), mmol/L | 4.95 (4.56, 5.57) | 4.88 (4.57, 5.23) | 5.04 (4.50, 6.25) | 0.163 |
| ALT, median (IQR), U/L | 17.00 (12.00, 27.00) | 17.00 (12.00, 27.00) | 17.00 (12.00, 27.00) | 0.979 |
| AST, median (IQR), U/L | 18.00 (15.00, 23.50) | 20.00 (16.00, 24.00) | 17.50 (15.00, 22.00) | 0.216 |
| Uric acid, median (IQR), µmol/L | 316.00 (257.00, 364.00) | 324.00 (274.50, 401.00) | 297.50 (243.00, 351.75) | 0.054 |
| Homocysteine, median (IQR), µmol/L | 14.10 (11.10, 17.10) | 14.40 (11.05, 16.65) | 13.85 (11.15, 17.93) | 0.640 |
| Cystatin C, median (IQR), mg/L | 1.03 (0.93, 1.15) | 1.00 (0.93, 1.09) | 1.05 (0.92, 1.18) | 0.225 |
| Complement c1q, mean±SD, mg/L | 160.32±24.91 | 161.88±23.58 | 158.72±26.37 | 0.552 |
| Serum potassium, mean±SD, mmol/L | 4.25±0.37 | 4.24±0.41 | 4.26±0.34 | 0.805 |

| | | | | |
|---|---|---|---|---|
| Serum sodium, median (IQR), mmol/L | 142.00 (140.50, 144.00) | 142.00 (140.50, 145.00) | 142.00 (140.25, 144.00) | 0.538 |
| Serum calcium, median (IQR), mmol/L | 2.28 (2.22, 2.34) | 2.27 (2.23, 2.35) | 2.28 (2.22, 2.33) | 0.930 |
| Serum magnesium, mean±SD, mmol/L | 0.87±0.71 | 0.85±0.07 | 0.88±0.07 | 0.096 |
| Creatine kinase, median (IQR), U/L | 63.00 (49.00, 82.00) | 64.00 (54.50, 87.00) | 62.00 (45.25, 78.00) | 0.182 |
| Creatine kinase isoenzymes, median (IQR), ng/L | 1.50 (1.10, 2.10) | 1.50 (1.00, 2.10) | 1.40 (1.10, 2.08) | 0.923 |
| Troponin I, median (IQR), ng/L | 9.64 (5.13, 25.68) | 9.87 (5.43, 29.76) | 9.21 (5.11, 23.56) | 0.712 |
| LDH, median (IQR), U/L | 209.00 (186.50, 246.50) | 219.00 (188.00, 266.50) | 203.50 (177.50, 239.75) | 0.256 |
| NT-proBNP, median (IQR), pg/ml | 407.00 (284.65, 719.55) | 375.60 (289.90, 806.10) | 445.00 (276.08, 654.65) | 0.976 |

Abbreviation: OPCABG, Off-pump Coronary Artery Bypass Grafting; LDL-C, Low-density lipoprotein cholesterol; HDL-C, High-density lipoprotein cholesterol; BUN, Blood Urea Nitrogen; ALT, Alanine transaminase; AST, Aspartate transaminase; LDH, Lactate dehydrogenase; NT-proBNP, N terminal pro B type natriuretic peptide.
