# Supplementary material for: Optimal postoperative delirium prediction after coronary artery bypass grafting surgery: a prospective cohort study
Source: Front Cardiovasc Med. 2023 Dec 8;10:1251617. doi: 10.3389/fcvm.2023.1251617 (PMC10739452; doi:10.3389/fcvm.2023.1251617)
Supplement: Supplementary file 2 [file Table2.pdf]

Supplementary table 2 Comparison of hemodynamic status and use of inotropes between two groups.

Variables	Low-ACCI score group (n=45)	High-ACCI score group (n=44)	*P* values
**Hemodynamic status**			
SBP during tracheal intubation (mmHg)	139.00(129.00, 156.50)	143.50(125.50, 165.00)	0.619
DBP during tracheal intubation (mmHg)	78.00 (64.50, 92.00)	70.00(65.00, 77.50)	0.102
MAP during tracheal intubation (mmHg)	99.00 (86.50, 113.50)	96.00(86.50, 105.75)	0.423
HR during tracheal intubation	83.00 (74.50, 96.00)	82.00 (69.25, 96.75)	0.616
SBP during sternotomy (mmHg)	119.00(110.50, 131.50)	127.50(117.00, 137.50)	0.085
DBP during sternotomy (mmHg)	64.00 (54.00, 75.00)	60.50(54.00, 65.75)	0.075
MAP during sternotomy (mmHg)	86.00 (75.00, 95.00)	83.50(78.00, 92.00)	0.666
HR during sternotomy	60.00 (51.50, 71.50)	60.00 (52.00, 68.00)	0.935
SBP after CABG (mmHg)	125.00(114.50, 139.50)	134.00(121.75, 145.75)	0.064
DBP after CABG (mmHg)	68.00 (56.00, 78.00)	64.50(56.00, 71.00)	0.136
MAP after CABG (mmHg)	88.00 (77.50, 99.50)	89.50(80.25, 96.75)	0.857
HR after CABG	64.00 (54.50, 74.00)	60.00 (54.00, 67.00)	0.182
**Inotropes, No (%)**			
Dopamine	6 (13.3)	5（11.4）	0.551
Adrenaline	3 (6.7)	4（9.1）	0.539
Norepinephrine	11 (24.4)	13（29.5）	0.180
Metaraminol	13 (28.9)	12（27.3）	0.137

Bold values indicated statistical significances. Data of hemodynamic status are presented as median (IQR).

Abbreviation: SBP, Systolic blood pressure; DBP, Diastolic blood pressure; MAP, Mean arterial pressure; HR, Heart rate; CABG, Coronary artery bypass grafting.
